# Experimental Investigation on Velocity and Temperature Field in a Rotating Non-isothermal Turbulent Boundary Layer using Hot-wire

**DOI:** 10.1038/s41598-020-66853-6

**Published:** 2020-06-18

**Authors:** Li Gangfu, Li Haiwang, You Ruquan, Wu Huijie, Tao Zhi, Xia Shuangzhi

**Affiliations:** National Key Laboratory of Science and Technology on Aero Engines Aero-thermodynamics, Aircraft/Engine Integrated System Safety Beijing Key Laboratory, Beihang University, Beijing, 100191 China

**Keywords:** Aerospace engineering, Fluid dynamics

## Abstract

This experiment measured the instantaneous temperature and velocity field synchronously in non-isothermal turbulent boundary layer in a rotating straight channel with a parallel-array hot-wire probe. The Reynolds number based on the bulk mean velocity (*U*) and hydraulic diameter (*D*) is 19000, and the rotation numbers are 0, 0.07, 0.14, 0.21 and 0.28. The mean velocity *u* and mean temperature *T* as well as their fluctuating quantity *u’* and *T’* were measured at three streamwise locations (*x/D* = 4.06, 5.31, 6.56). A method for temperature-changing calibration with constant temperature hot-wire anemometers was proposed. It achieved the calibration in operational temperature range (15.5 °C–50 °C) of the hot-wire via a home-made heating section. The measurement system can obtain the velocity and temperature in a non-isothermal turbulent boundary layer at rotating conditions. The result analysis mainly contains the dimensionless mean temperature, temperature fluctuation as well as its skewness and flatness and streamwise turbulent heat flux. For the trailing side, the rotation effect is more obvious, and makes the dimensionless temperature profiles lower than that under static conditions. The dimensionless streamwise heat flux shows a linear decrease trend in the boundary layer. It is hoped that this research can improve our understanding of the flow and heat transfer mechanism in the internal cooling passages of turbine rotor blades.

## Introduction

The development of internal cooling techniques have played an important role in allowing turbine rotor blades to work well in the environment above their melting point for the life of design. J. C. Han and his colleagues^1,2^ summarized the overall demand, development direction and the classic structure of this field in recent decades with many specific engine models, such as F117, GE CF6, etc. They also summarized the most important research contributions on turbine blade internal cooling studies at Texas A&M University’s Turbine Heat Transfer Laboratory. Among these techniques, serpentine passage in the middle section of a turbine blade has been investigated for more than thirty years. The increasing demands of higher efficiencies of gas turbine engines require more efficient techniques.

However, limited by technical difficulties under rotating and heating conditions, most of the experimental investigations could only measure flow field or heat transfer separately. Comparing with the mean quantities, the internal details especially the fluctuating quantities are more challenging for measurement. Since flow and heat transfer are coupled and influence each other, measuring one of them separately is not conducive to investigating their influence on each other. Generally speaking, rotating and heating conditions generate centrifugal force, Coriolis force and buoyancy force. This makes the principle more complex than isothermal static conditions. Therefore, this experiment measured the instantaneous temperature and velocity field synchronously in non-isothermal turbulent boundary layer in a rotating channel with hot-wire anemometry system.

Plenty of investigations in rotating ducts have been carried out via different techniques by a variety of researchers in last few decades. The researches on flow without heating mainly focused on the mean velocity profiles of developing or fully developed turbulent flow, secondary flow and the effects of rotation on the flow fields in a channel with complex geometry, such as U turns and ribs.

Koyama *et al*.^3^ studied the influence of the Coriolis force on the developing turbulent boundary layer in a rotating channel with hot-wire. He found that the secondary flow caused by the Coriolis force would make the leading side more stable and the trailing side less stable (The Coriolis force points from the leading side to the trailing side. This can be used to distinguish the two sides of a rotating duct). The effects on other two sides could be ignored. Maciel *et al*.^4^ studied the fully developed turbulent boundary layer characteristics in a rotating channel with hot-wire. The experimental conclusion is that the development of the turbulent boundary layer appears to take two forms: standard two-dimensional flow development and accelerated flow due to the development of end wall effects. Wagner *et al*.^5^ studied the secondary flow in a rotating channel with pitot tube. The research showed that the cross flow velocity and the longitudinal vorticity were linearly proportional to the rotation number. The influence of pressure on the flow field is also involved. Brossard *et al*.^6^ studied a rotating U-shaped smooth channel with 60° parallel ribs inside with PIV (Particle Image Velocimetry). Although these PIV measure-ments were obtained without buoyancy effects, they could be used for comparison with results from numerical simulations. Wei *et al*.^7^ analyzed the effect of the rotation numbers and the streamwise positions on the dimensionless velocity profiles. In addition, the critical rotation number phenomenon, the peak variation of the velocity pulsation, the influence depth of the Coriolis force and the relaminarization process are also discussed.

As for local heat transfer experimental investigations, J. H. Wagner and his colleagues^8–10^ have made an outstanding contribution. There are mainly four parameters influencing heat exchange effect in rotating passages: density ratio ($({T}_{w}-{T}_{b})/{T}_{w})$, rotation number ($\Omega D/U$), Reynolds number $(\rho UD/\mu )$ and ratio of rotation radius to hydraulic diameter (*r/D*). They showed that these parameters need to be mainly controlled in heat transfer experimental studies in this field. The results comparison showed that heat transfer was strongly influenced by rotation. Under smooth wall conditions, the increase in heat transfer at the trailing side reached 3.5 times that of the fully developed boundary layer and the heat transfer at the leading side decreases to 40%. When the wall is ribbed, the Nusselt number ratio is more complicated by the influence of buoyancy number and density ratio on the leading side of the rotating channel.

The influence of thermal boundary conditions on heat transfer and flow is also worth studying. Parsons *et al*.^11^ investigated the local heat transfer under three different thermal boundary conditions in a ribbed U channel. The experimental results showed that under three different thermal boundary conditions, the heat transfer of the ribbed U channel was the worst when the same temperature was maintained on the four surfaces. The reason is that the secondary flow caused by rotation can promote fluid at different temperatures mixing and enhance heat transfer. But maintaining the same temperature on each wall will reduce this effect. In addition, the study found that the rotation number, Reynolds number and buoyancy parameter can also have a significant effect on the Nusselt number ratios (*Nu*/*Nu*_0_). Here, *Nu* represents local Nusselt number (*hD/*$\lambda ,$ *h* represents heat transfer coefficient) and *Nu*_0_ represents Nusselt number in fully developed turbulent tube flow. You *et al*.^12^ studied heat transfer when one, two or four walls of a square rotating channel were heated. The variation of Nusselt number ratios caused by three heating conditions can reach ten percent. To explain this phenomena, secondary flow in the rotating channel was measured by PIV, which indicates that the design of the internal cooling channel also needs to consider thermal boundary conditions.

The main focus of the above heat transfer researches is Nusselt number (or heat transfer ratio). Details on the velocity and temperature field inside the rotating channel are limited due to different focuses. In order to obtain reliable internal data, it is necessary to develop techniques for measuring velocity and temperature field simultaneously. In M. Tagawa^13^’s opinion, the synchronous measurement technology can be roughly divided into two types: contact and non-contact types. Although non-contact measurement does not interfere with the measurement field, its equipment is often more complex and larger in size. It would be very difficult to extend the static experimental investigation into that under rotating conditions. Namely, non-contact types seem to be in a developing stage currently, especially for temperature measurement. M. Tagawa^14^ combined LDV (Laser Doppler Velocimetry) and digitally compensated fine-line thermocouples to achieve simultaneous velocity and temperature measurement. They measured the turbulence near the heating cylinder to verify its reliability and showed the system would be suitable for a wide range of velocity and temperature.

Hot-wire technology has also become a practical system, because it is easier to add temperature measurement function among three higher-precision flow field testing techniques (Hot-wire, PIV, LDV). Adding a temperature measuring hot-wire to the original velocity measuring function hot-wire can achieve the purpose of measuring velocity and temperature simultaneously. However, there is no such commercially available hot-wire currently. Blair *et al*.^15^ and Bagheri *et al*.^16^ both utilized an x probe and a temperature probe, which are fixed on the same probe support to investigate heat transfer and flow field in stationary conditions. Since the signal of the probe will be affected by both velocity and temperature of the field, the calibration under non-isothermal conditions needs corresponding adjustment compared to that under isothermal conditions. Blair *et al*.^15^ described the development of an instrumentation system (probe design, system design, probe calibration process, etc.) designed for the simultaneous measurement of temperature and velocity field. The author believed that the hot-wire technology is still the most practical technology currently for measuring velocity and temperature at the same time, as well as their fluctuations. Well, Bagheri *et al*.^16^ mainly focused on the application of technology and measured Prandtl numbers in turbulent boundary layer. The turbulent Prandtl number $P{r}_{t}$ is about 0.9 for air when there is no streamwise pressure gradient. But it has not a constant value and varies with the positions in the turbulent boundary layer and the nature of the boundary layer. For three temperature difference cases (the temperature differences were 10 °C, 15 °C, 20 °C), the turbulent Prandtl numbers were indeed different, but they had a consistent change trend and were basically within the uncertainty envelope of previous studies. In heat transfer research, dimensionless mean temperature ${T}^{+}\left(=\frac{{T}_{w}-T}{{T}_{\tau }}\right)$ is widely used, which is a good parameter for analyzing heat transfer details. Houra *et al*.^17^ studied the effects of adverse pressure gradient on dimensionless mean temperature in thermal boundary layer under static conditions, which does not involve the influence of the rotation effects on it.

As a summary, researchers have investigated heat transfer and flow field in internal cooling channels from different focuses in previous studies. However, internal details of the channel on the velocity and temperature field under different conditions are limited, especially the simultaneous measurement of velocity and temperature distribution. In particular, the experimental investigation on turbine blade internal cooling technology needs to meet the non-isothermal condition, high rotational speed, developing turbulent boundary layer, etc. Taking into account the advantages of hot-wire mentioned above and its convenience to work on a rotating test bench, a hot-wire anemometry system was developed to measure the instantaneous temperature and velocity field synchronously in non-isothermal turbulent boundary layer in a rotating channel. A new method for temperature-changing calibrations with home-made constant temperature hot-wire anemometers was proposed. By comparing with previous data and standard law of the wall (dimensionless mean velocity and mean temperature), it indicates that the experimental results are credible. The result analysis mainly contains the dimensionless mean temperature ${T}^{+}$, temperature fluctua-tion as well as its skewness and flatness and streamwise turbulent heat flux. Some phenomena and disciplines will be described in details later. It is hoped that this research can improve our understanding of the flow and heat transfer mechanism in the internal cooling passages of turbine rotor blades.

## Experimental Facility and Design

### The rotary test bench

**Figure 1:**
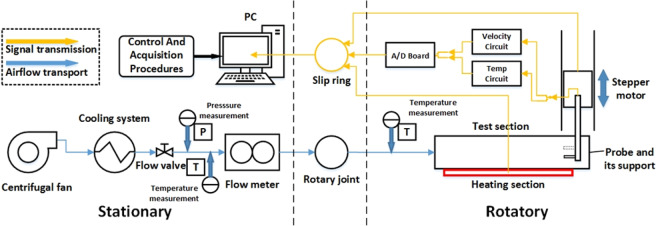
Schematic diagram of the rotating facility.

The general arrangement of the rotating facility is shown in Fig. 1. The main part of the facility has been introduced in our previous work^7^. The composition and working mode of the rotating experimental facility will be briefly introduced herein. The centrifugal fan draws air directly from the room as the coolant. In order to control the inlet conditions of the air, a cooling system is applied to control inlet air temperature. The air then flows through a flow meter and finally enters the experiment section through a rotary joint. The experimental control signals and data measurement signals are transmitted to a computer through multiple slip rings. The rotating part of the test rig is fixed on a disk with a vertical axis of rotation driven by a motor. The rotary test bench design has a maximum radius of about 1 m and a maximum rotational speed of 500 rpm.

### The square channel of the test section

**Figure 2:**
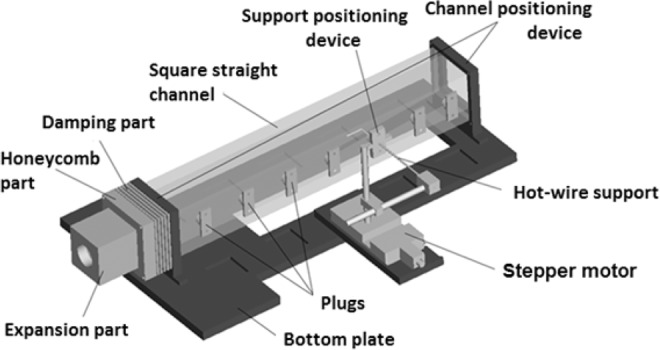
The square channel of the test section.

As mentioned above, this experimental research works on turbine blade internal cooling technology. Therefore, the design of the test section should be consistent with the internal cooling passage of a turbine blade. The square channel of the test section is shown as Fig. 2. The adjusting section is used to smooth the air before it entering to the test section, which mainly consists of expansion part, honeycomb part and damping part. The inlet turbulence intensity of the inlet air is about 1%. To simulate the thermal boundary conditions of the channel, ITO (Indium Tin Oxide) heater glass is pasted on the inner side of the 15-mm thick plexiglass channel wall and its surface is very smooth. It can conduct electricity to heat the flow.

**Figure 3:**
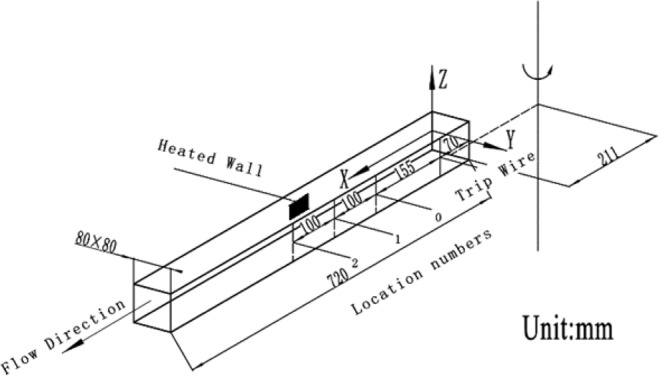
The geometry of the square channel.

In the experiment, the origin of the coordinate system is at the midpoint of the inlet of the heating wall surface as shown in Fig. 3. *x*, *y*, *z* represent for streamwise coordinate, normal coordinate and spanwise coordinate, respectively. The heated wall is located on the *x-z* plane. The small black square marks the location of it. The whole length of the wall is heated during the experiment. The distance between the origin of the coordinate system and the vertical axis of rotation is 211 mm.

The experimental parameters of the square channel is shown in Table 1. The Reynolds number $(\rho UD/\mu )$ is based on bulk average velocity *U*. The hydraulic diameter of the channel *D* is 80 mm. The rotation number *Ro* is defined as $\Omega D/U$. ${q}_{w}$ represents for wall heat flux.

**Table 1:** Experimental parameters of the square channel.

*Re*	19000
*Ro*	0, 0.07, 0.14, 0.21, 0.28
*x*/*D*	4.06, 5.31, 6.56
*q*_*w*_	380 W/*m*^2^ ± 6.4%

### Hot wire anemometry

#### Anemometry and hot-wire probe

**Figure 4:**
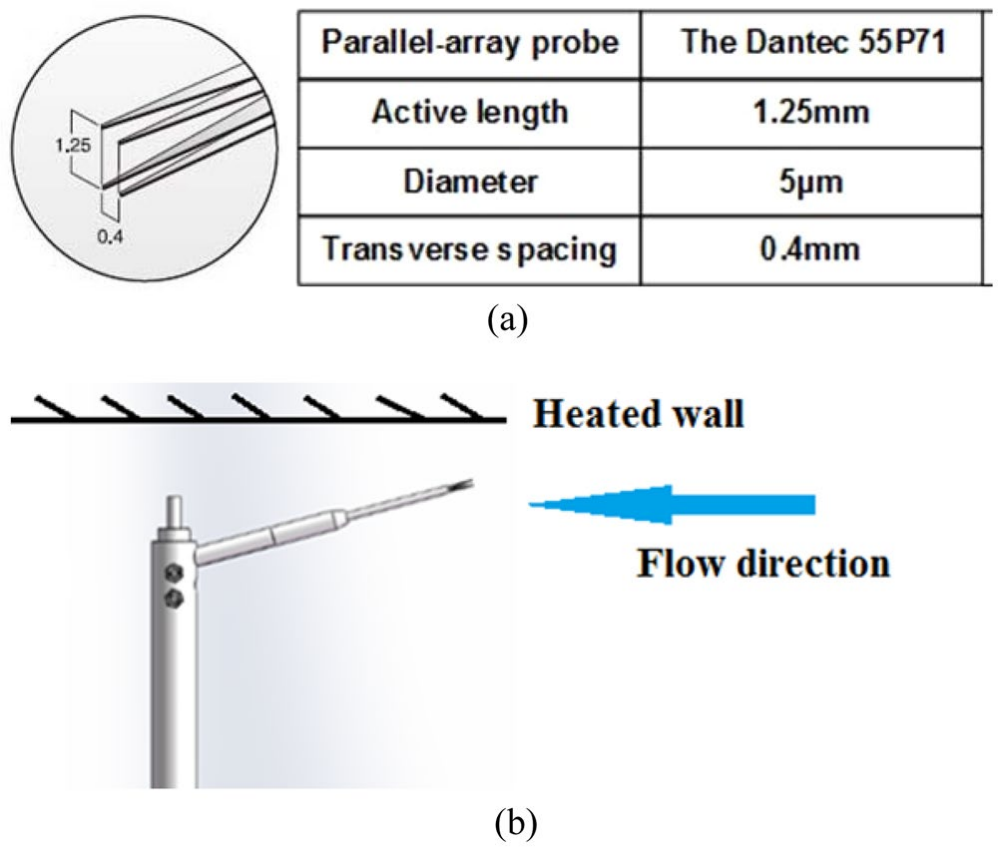
The parallel-array probe. (**a**) The main parameters of the probe; (**b**) The arrangement of the probe and its support.

The hot-wire probe has a very high spatial resolution in the process of measurement. Therefore, it is possible to measure the change of the large gradient velocity and temperature near the wall in the turbulent boundary layer accurately. In this research, The Dantec 54T42 Mini Constant Temperature Anemometry (MiniCTA) is utilized with the Dantec 55P71 parallel-array probe and a home-made temperature measuring circuit. The main parameters of the probe are shown in Fig. 4a. The overheat ratio of the probe is 1.58 in the experiment process. The data acquisition frequency $f$ of the probe is 2000 Hz. The research by Hutchins N *et al*.^18^ suggested three guidelines to guarantee hot-wire spatial resolution on wall-bounded turbulent experiments: (a) ${l}^{+}\left(=\frac{l{u}_{\tau }}{\nu }\right)$ < 20. (b) *l/d* ≥ 200. (c) ${f}^{+}\left(=\frac{f\nu }{{{u}_{\tau }}^{2}}\right)$ > 1*/*3. According to the values in Fig. 4a, *l/d* = 1.25 mm/5 μm = 250 > 200. Due to the slight difference of ${u}_{\tau }$ and $\nu$ under different working conditions, one set of working conditions is taken for calculation ($({u}_{\tau }=0.220\,m/s\,{\rm{and}}\,\nu =1.657\times {10}^{-5}\,{m}^{2}/2)$. In this situation, ${l}^{+}=16.6 < 20$ and ${f}^{+}=0.68$ >1*/*3. It can be seen that this experiment fits three guidelines of Hutchins N^18^ well.

According to the basic principle of hot-wire technology, to maintain the constant temperature of a hot-wire probe, it is necessary to increase the voltage drop across the hot-wire probe to compensate for the cooling of the wire by the fluid flow. The balance voltage is mainly affected by the velocity and temperature of fluid. Therefore, it is necessary to obtain the temperature of the flow field to determine the velocity with the balance voltage. The advantage of using the other hot-wire of a parallel-array hot-wire to measure temperature signals is that it has not to introduce a new set of equipment. Namely, this arrangement could simplify the experimental setup. Similar to other researches in the same field^13–16^, this hot-wire acts like a temperature sensitive resistance. However, its measurement of temperature is affected by changes in velocity, which makes it different from conventional temperature sensitive resistance. In our previous article^19^, the arrangement of the probe has been introduced in details as shown in Fig. 4b. It also proved that the probe can be used to measure the temperature.

#### Calibration of hot-wire technology

**Figure 5:**
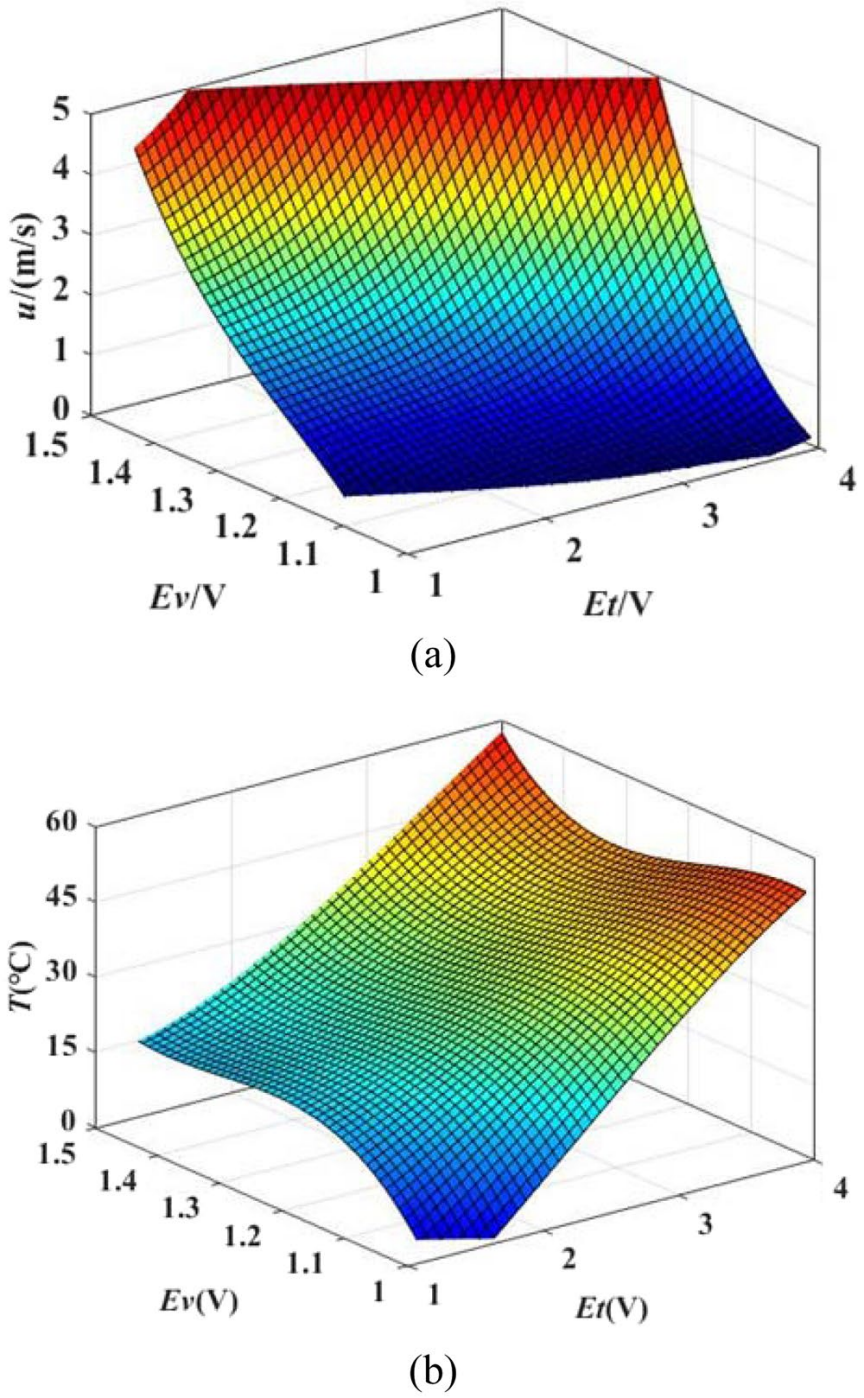
The calibration surfaces. (**a**) The calibration surface of *u*; (**b**) The calibration surface of *T*.

In the calibration process, a new method for temperature-changing calibration with home-made constant temperature hot-wire anemometers was proposed. StreamLine Pro automatic calibrator of Dantec Dynamics Company has high accuracy for velocity calibration under isothermal conditions. For a good dedicated calibrator, the uncertainty of the airflow velocity at the nozzle outlet is given in the user’s manual as ±1% ± 0.02 m/s. However, this commercial calibrator does not provide temperature-changing functions. Considering this situation, adding a heating section to the calibrator is a more economical and convenient solution than redesigning a wind tunnel for present research. Therefore, a heating section based on the original calibrator was designed. It consists of a heating wire and ceramic, which can control the temperature of the wind tunnel outlet by changing the voltage of the heating wire. With the modified calibrator, the final velocity and temperature calibration range are 0.15 m/s ~ 5 m/s and 15.5 °C (room temperature) ~50 °C, respectively. This study uses the method of surface fitting so that the explicit forms of relationships between the required physical quantities and the direct measurement quantities are not necessary. The calibration surfaces of velocity and temperature are shown as Fig. 5. *Ev* and *Et* are the voltage signal values directly measured by the velocity circuit and the temperature circuit, respectively. Two surfaces in Fig. 5a,b are fitted by 180 calibration points. It can be seen from the figure that the fitting surfaces are very smooth and have no overlapping parts.

### Uncertainty analysis

The main sources of uncertainty in this study come from the device’s system uncertainty, conditional control uncertainty and data fitting uncertainty. The direct measurement quantities in this research are temperature and velocity. During the calibration process, the uncertainty of Omega T-type thermocouple which monitored the temperature of nozzle outlet is about ±0.2 °C. Since the thermocouple cannot be spatially fully coincident with the hot-wire probe, the resulting control uncertainty is approximately ±0.3 °C. The data fitting uncertainty can be divided into two parts: the surface fitting uncertainty of the calibration data and the fitting uncertainty caused by the spatial distance between two hot-wire probes. The total uncertainty caused by the above two sources is ±0.3 °C. This part of the error can be statistically obtained from the calculation program results. Considering all uncertainty sources mentioned above, the uncertainty of the temperature measured with the hot wire anemometry system is ±0.8 °C.

As for the velocity, the uncertainty of the airflow velocity at the nozzle outlet is given in the user’s manual for the present calibrator as ±1% ± 0.02 m/s. In addition, the surface fitting uncertainty of velocity calibration data is ±0.02 m/s. According to the user’s manual, the relative uncertainty of velocity caused by temperature-induced density change is $\Delta T/(\sqrt{3}\cdot 273)$. The uncertainty of this part is about ±0.2%. Therefore, the total uncertainty of velocity is ±1.2% ± 0.04 m/s.

It is more convenient to have dimensionless quantities normalized by wall units in the analysis of results. The wall quantities utilized for dimensionless are mainly friction velocity ${u}_{\tau }$ and friction temperature ${T}_{\tau }$ which calculation formulas are given by (1) and (2).

1 $${u}_{\tau }=\sqrt{{\tau }_{w}/\rho }=\sqrt{{\left(\mu \frac{\partial u}{\partial y}\right)}_{w}/\rho }=\sqrt{(\mu k)/\rho }$$

2 $${T}_{\tau }=\frac{{q}_{w}}{\rho {c}_{p}{u}_{\tau }}$$

For Eq. (1), the viscous sublayer profile method^20^ is used to obtain the wall shear stress ${\tau }_{w}$, which is a very important flow characteristic parameter in turbulent boundary layer. *k* is the velocity gradient in the viscous sublayer. Its error (the maximum value is about 9%) is directly obtained by statistics. Through the analysis of the data results, it can be seen that the linearization degree of the velocity in the recommended range of the study^20^ is very high in this experiment. Therefore, this method of determining the distance between the measuring point to the wall and the wall shear stress is still effective. As for (2), constant heat flow boundary conditions are adopted and the heat flux ${q}_{w}$ is about 380 W/${m}^{2}$. This value has a slight change under different experiment conditions. The error on the heat flux is about 6.4%. The errors on friction velocity and friction temperature are calculated as follows. Since the variable physical parameters are used in the calculation, the uncertainty generated in this part is relatively small and neglectable.

3 $$\frac{\Delta {u}_{\tau }}{{u}_{\tau }}=\left(\sqrt{{\left(\frac{\partial {u}_{\tau }}{\partial k}\right)}^{2}\cdot {(\Delta k)}^{2}}\right)/{u}_{\tau }=\frac{1}{2}\cdot \frac{\Delta k}{k}=4.5 \% $$

4 $$\frac{\Delta {T}_{\tau }}{{T}_{\tau }}=\left(\sqrt{{\left(\frac{\partial {T}_{\tau }}{\partial {q}_{w}}\right)}^{2}\cdot {(\Delta {q}_{w})}^{2}+{\left(\frac{\partial {T}_{\tau }}{\partial {u}_{\tau }}\right)}^{2}\cdot {(\Delta {u}_{\tau })}^{2}}\right)/{T}_{\tau }=7.8 \% $$

With these two wall parameters, the dimensionless parameters of velocity and temperature can be obtained. The calculation formulas and the error transfer formulas are shown as follows. The approximate uncertainties of several main calculation quantities are given in the form of Table 2.

5 $${y}^{+}=\frac{{u}_{\tau }y}{\nu }\,{u}^{+}=\frac{u}{{u}_{\tau }}\,{T}^{+}=\frac{{T}_{w}-T}{{T}_{\tau }}$$

6 $$\frac{\Delta {y}^{+}}{{y}^{+}}=\left(\sqrt{{\left(\frac{\partial {y}^{+}}{\partial y}\right)}^{2}\cdot {(\Delta y)}^{2}+{\left(\frac{\partial {y}^{+}}{\partial {u}_{\tau }}\right)}^{2}\cdot {(\Delta {u}_{\tau })}^{2}}\right)/{y}^{+}=4.6 \% $$

7 $$\frac{\Delta {u}^{+}}{{u}^{+}}=\left(\sqrt{{\left(\frac{\partial {u}^{+}}{\partial u}\right)}^{2}\cdot {(\Delta u)}^{2}+{\left(\frac{\partial {u}^{+}}{\partial {u}_{\tau }}\right)}^{2}\cdot {(\Delta {u}_{\tau })}^{2}}\right)/{u}^{+}=4.9 \% $$

8 $$\frac{\Delta {T}^{+}}{{T}^{+}}=\left(\sqrt{{\left(\frac{\partial {T}^{+}}{\partial T}\right)}^{2}\cdot {(\Delta T)}^{2}+{\left(\frac{\partial {T}^{+}}{\partial {T}_{\tau }}\right)}^{2}\cdot {(\Delta {T}_{\tau })}^{2}}\right)/{T}^{+}=8.8 \% $$

**Table 2:** Uncertainties of the main parameters.

${u}_{\tau }$	4.5%
${T}_{\tau }$	7.8%
*y*^+^	4.6%
*u*^+^	4.9%
*T*^+^	8.8%

### Verification of techniques

Before the analysis of the results, verification of technical reliability is necessary. By comparing with previous study, the results of present work will be proved to be credible. For parallel-array hot-wire probe, the first thing to verify is the influence between the two hot-wire probes. This part of the verification process has been detailed in our previous article^19^. It suggests that the impact of the two hot-wire probes is acceptable and the apparatus has high spatial resolution in the measurement process. The three parameters of the probe (−*l*^+^, *l/d* and *f*^+^) also satisfy three guidelines of Hutchins N *et al*.^18^ as shown in Section 2.3.1.

#### Dimensionless velocity and temperature contrast with previous work

**Figure 6:**
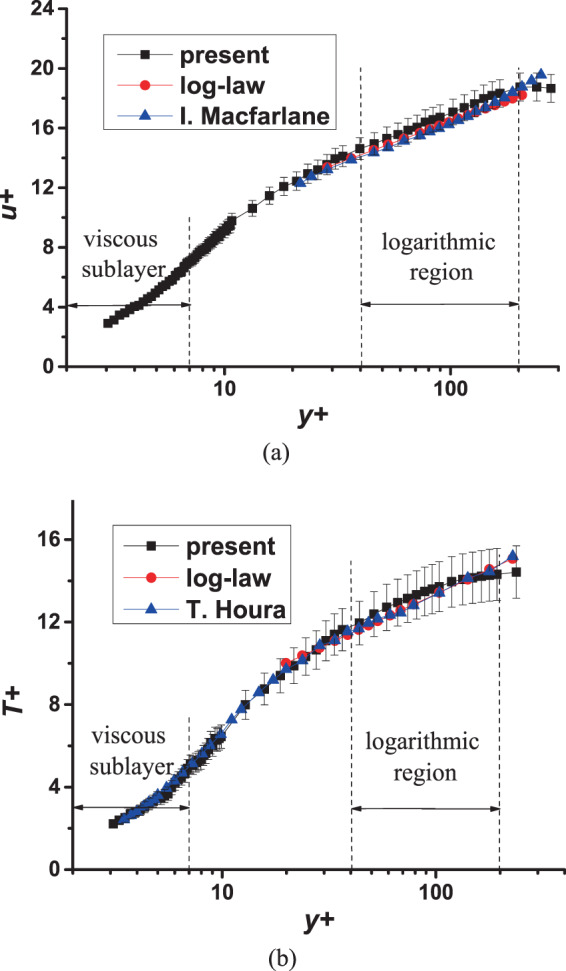
Verification of techniques. (**a**) Comparison of *u*^+^ with I. Macfarlane^21^; (**b**) Comparison of *T*^+^ with T. Houra^17^.

The mean profiles of dimensionless velocity ${u}^{+}$ and dimensionless temperature ${T}^{+}$, which are defined in formula (5) are compared with previous work^17,21^, respectively. The comparison results are shown in Fig. 6a,b. The measurements are performed at location 1 (*x/D* = 5.31) with *Re* = 19000 and *Ro* = 0. Since the distributions of ${u}^{+}$ and ${T}^{+}$ of the three measuring locations under static conditions are basically the same, only the curves measured at location 1 are shown.

The log-law formula in Fig. 6a is ${u}^{+}=2.44\,\mathrm{ln}\,{y}^{+}+5.2$^21^. It should be noted that the contrast in this figure is under unheated conditions. According to the previous calculation, the error band is taken as 4.9%. It can be seen from the figure that the error band can completely overlap the standard log-law and the results of previous work. When ${y}^{+}$ is greater than 200, the ${u}^{+}$ change of the current results is relatively small but the ${u}^{+}$ of^21^ continues to increase. This is understandable because the boundary layer in this study is developing boundary layer. Since the degree of development is not high, the boundary layer is relatively thin.

The log-law formula in Fig. 6b is ${T}^{+}=2.075\,\mathrm{ln}\,{y}^{+}+3.8$^17^. According to the previous calculation, the error band is taken as 8.8%. It can be seen that the dimensionless velocity ${u}^{+}$ and the dimensionless temperature ${T}^{+}$ in the logarithmic region are both slightly higher than the contrast value. According to the study^22^, the positive pressure gradient or the adverse pressure gradient will cause the mean profiles to be higher or lower than the standard value of the logarithmic zone which is under zero pressure gradient. The data from the current experiment in both figures are slightly higher than the comparison values, indicating that there is a very slight positive pressure gradient in the test. Although this experiment did not accurately measure the streamwise positive pressure gradient in the channel, the flow meter did detect that the channel inlet pressure (about 104 kPa) was slightly greater than the environment pressure (about 101 kPa). This verifies the previous prediction. Similar to the trend of ${u}^{+}$, the latter part of the logarithmic region of ${T}^{+}$ has a slower growth trend, which is also due to the low degree of development of the boundary layer.

In summary, the agreement between the present results and the previous results can demonstrate the reliability of the results in the present work.

#### Development of velocity and temperature boundary layer

**Figure 7:**
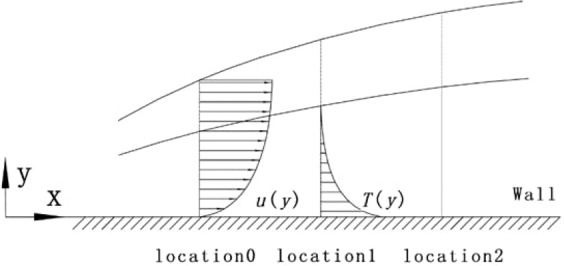
Schematic diagram of the velocity and thermal boundary layers.

As mentioned above, the degree of development of the turbulent boundary layer in this study is not high. Figure 7 shows the degree of development of the boundary layer under static conditions in this study. The velocity and thermal boundary layer thickness in the figure are the momentum thickness ${\delta }_{2}$ and enthalpy thickness ${\Delta }_{2}$ defined in formula (9) and (10). Subscript $\infty$ represents the mainstream. The specific values of the velocity boundary layer thickness of the three streamwise locations are 1.4099 mm, 1.8282 mm, 2.1061 mm. As for the thermal boundary layer thickness, the specific values of the three streamwise locations are 0.8816 mm, 1.1440 mm, 1.3391 mm. The velocity boundary layer of this experiment is slightly thicker than the thermal boundary layer under static conditions.

9 $${\delta }_{2}={\int }_{0}^{\infty }\frac{\rho u}{{\rho }_{\infty }{u}_{\infty }}\left(1-\frac{u}{{u}_{\infty }}\right)dy$$

10 $${\Delta }_{2}={\int }_{0}^{\infty }\frac{\rho u}{{\rho }_{\infty }{u}_{\infty }}\left(\frac{T-{T}_{\infty }}{{T}_{w}-{T}_{\infty }}\right)dy$$

It can be seen that the boundary layer is very thin compared to hydraulic diameter of the channel *D* (80 mm). Taking previous research^17^ for comparison, the thickness of velocity boundary layer can reach 30 mm, which is much thicker than that in current work. This is because that the rotating test bench is difficult to carry an excessively long channel. On the other hand, the boundary layer of the internal cooling channel is not fully developed boundary layer. The final aim is to make the conditions of this experimental study as close as possible to the working conditions of the internal cooling passage in the turbine rotor blade.

## Results and discussion

Before presenting the experimental results, the theoretical analysis of flow and heat transfer under rotating coordinate system should be introduced. For the study of internal cooling channels in turbine rotor blades, the rotating coordinate system is more convenient. Since the rotating coordinate system is a non-inertial system, the standard form of the N-S equation is no longer applicable. The incompressible fluid turbulence control equations in the constant rotational speed rotation system is as follows:

11 $${\rm{Continuity}}\,{\rm{equation}}:\nabla \cdot (\rho \overrightarrow{v})=0$$

12 $${\rm{Momentum}}\,{\rm{equation}}:\rho (\overrightarrow{v}\nabla \rangle \overrightarrow{v}=-\nabla {p}_{eff}+(\mu +{\mu }_{t}\rangle {\nabla }^{2}\overrightarrow{v}-\rho (2\overrightarrow{\varOmega }\times \overrightarrow{v}+\overrightarrow{\varOmega }\times \overrightarrow{\varOmega }\times \overrightarrow{r}\rangle $$

13 $${\rm{Energy}}\,{\rm{equation}}:\rho {c}_{p}(\overrightarrow{v}\cdot \nabla T)=(\lambda +{\lambda }_{t}){\nabla }^{2}T$$

Here, ${p}_{eff}$ is a combined pressure value, including static pressure and contribution of centrifugal force field. ${\mu }_{t}$ and ${\lambda }_{t}$ are turbulent dynamics viscosity coefficient and turbulent thermal conductivity, respectively. It can be seen from Eq. (12) that the rotating coordinate system directly produces Coriolis force and centrifugal force, that is, the last two terms of the equation. The equations will become more complicated considering the nature of turbulent pulsation.

The above is the analysis without considering the density change. Since the density ratio *d.r*., which is defined as $({T}_{w}-{T}_{b})/{T}_{w}$, is about 0.1 in current experiment, the effect of density change cannot be ignored. Considering the effect of temperature gradient on density inhomogeneity, the density linearization can be linearized as:

14 $$\rho ={\rho }_{0}[1-\gamma (T-{T}_{0})]={\rho }_{0}[1-\gamma \Delta T]$$

Here, $\gamma$ is the volume expansion coefficient of the cool gas, which is considered as 1/*T* for the perfect gas. ${T}_{0}$ is the reference temperature and ${\rho }_{0}$ is the density under ${T}_{0}$.

In addition to the analysis of the governing equations, the boundary condition or wall parameter also requires attention. For the flow field, an important parameter is the wall shear stress:

15 $${\tau }_{w}={\left(\mu \frac{\partial u}{\partial y}\right)}_{w}$$

Since the fluid near the wall is mainly affected by the viscosity, this part of the fluid has no relative flow to the wall. This is also called non-slip boundary condition. The viscous sublayer profile method^20^ which is mentioned above for determining the distance between the probe and the wall is based on this. The heat transfer between the wall and the fluid must pass through the thin viscous layer of fluid close to the wall. In the case of ignoring thermal radiation, the amount of heat transfer is equal to the amount of heat conduction of this thin layer of fluid very close to the wall. Therefore, the heat flux which is very important for the temperature field is calculated by:

16 $${q}_{w}={\left(-\lambda \frac{\partial T}{\partial y}\right)}_{w}$$

It should be noted that shear stress and heat flux at the point of complete coincidence with the wall cannot be obtained. But due to the high spatial resolution of the hot-wire probe, velocity and temperature fields which are very close to the wall can be utilized to calculate these two quantities. The linearization degree of data in this area is high. Figure 6 of the Verification of techniques section already shows that this method is reliable. In addition to the method of calculating the heat flux with the temperature field, direct measurement method can also be used. A measurement method was introduced in^12^. Although the direct measurement method is more accurate for the measurement of the heat flux itself, it is difficult to ensure that the boundary conditions are completely consistent for the introduction of TLCs (Thermographic Liquid Crystal) or other techniques for this experiment. Therefore, no direct measurement of heat flux is adopted.

### Distributions of dimensionless mean temperature profiles

As mentioned in the introduction, the focus of this paper is the effects of rotation on the details of internal cooling channel. These details can help understand flow and heat transfer mechanisms while also providing validation for numerical simulations. In general, the centrifugal force is proportional to the radius of rotation and the square of the rotational speed, while the Coriolis force is proportional to the rotational speed and flow velocity. Therefore, a larger rotation number *Ro* and a larger streamwise position *x/D* will make the forces stronger. In general, the rotation effects lead to velocity profiles loss near the leading side, which is the contrary near the trailing side^7^. This trend is more pronounced at downstream positions and high rotation numbers. From the dimensionless velocity profiles of his research results, it can be seen that the ${u}^{+}$ of the leading side is generally higher than that under static conditions and the ${u}^{+}$ of the trailing side is generally lower than that under static conditions. So what is the rotation effect on ${T}^{+}$?

**Figure 8:**
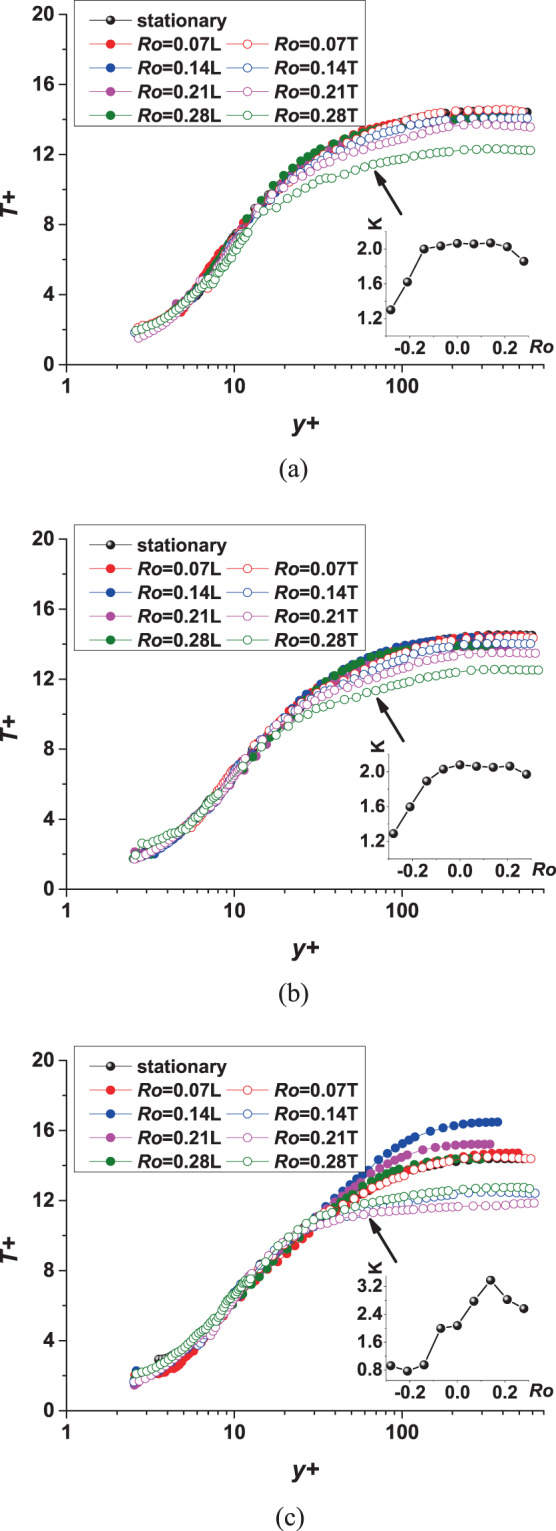
Dimensionless temperature profiles under different *Ro* and *x/D*. (**a**) location 0; (**b**) location 1; (**c**) location 2 (“L” represents for leading side and “T” represents for trailing side).

The rotation effect on ${T}^{+}$ in the case of *Re* = 19000 is shown in Fig. 8. For better observation, the slope K of the logarithmic region is added in the right corner of the graphs. In order to distinguish the data near leading side and trailing side, we define the rotation number of the leading side and trailing side to be positive and negative, respectively. The degree of influence of *Ro* and *x/D* on dimensionless temperature can be seen from these figures. For the leading side in Fig. 8a,b, rotation has little effect on dimensionless temperature ${T}^{+}$ when *x/D* = 4.06 or 5.31 (location 0 or location 1). The slopes of the logarithmic region are very close to the static standard value of 2.075. This shows that the use of friction temperature ${T}_{\tau }$ is a good way to standardize temperature. This parameter contains the information of heat flux and friction velocity as shown in Eq. (2). When *x/D* = 6.56(location 2) at the downstream in Fig. 8c, the ${T}^{+}$ with different *Ro* shows a significant difference after ${y}^{+}$ is greater than 30. As the *Ro* increases, the ${T}^{+}$ of the core region increases first and then decreases. The largest difference can reach 14.6%. This is similar to the critical rotation number phenomenon of the leading side velocity profiles in our previous study^19^. The influence of Coriolis force at the downstream will be weakened under certain circumstances. As for the trailing side, the rotation effect is more obvious and makes the dimensionless temperature profiles lower than that under static conditions. When *Ro* = 0.07, the dimensionless temperature profiles at the three positions is almost the same as the static state. This shows that when the rotation number is small, the rotation effect near the trailing side can be ignored. When *x/D* = 6.56(location 2) at the downstream in Fig. 8c, rotation makes the dimensionless temperature profiles significantly reduced and the maximum difference can reach 17.3%.

### Distributions of the statistics of the fluctuating temperature

#### Fluctuating temperature statistics

**Figure 9:**
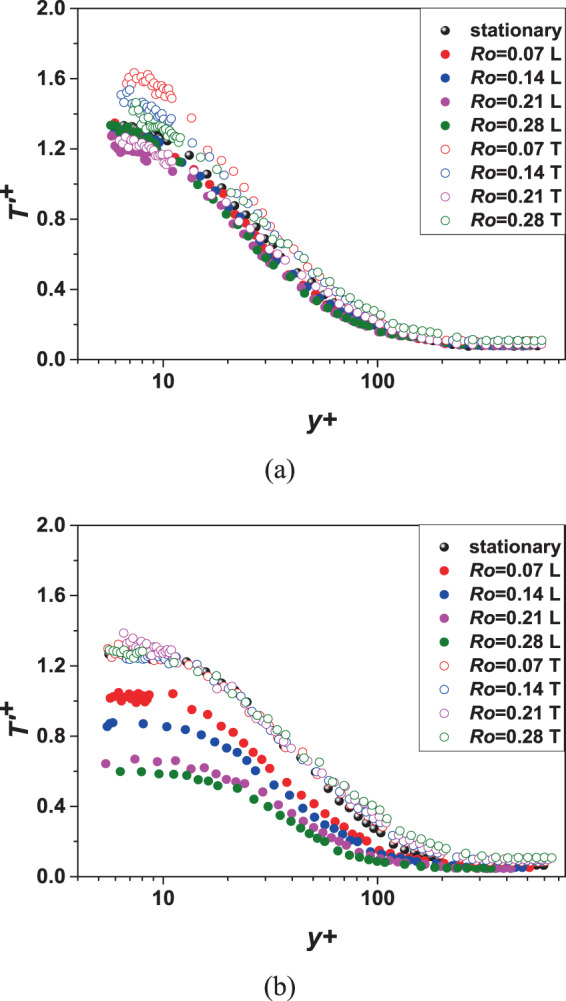
Fluctuating temperature statistics under different *Ro* and *x/D*. (**a**) location 0; (**b**) location 1.

Fig. 9 shows the variation of temperature fluctuations at location 0 and location 1 (*x/D* = 4.06, 5.31) with different *Ro*. The ordinate ${T{\prime} }^{+}\left(=\sqrt{\frac{\overline{T{\prime} T{\prime} }}{{{T}_{\tau }}^{2}}}\right)$ can reflect the intensity of temperature fluctuations. In these figures, letter “L” represents for leading side and letter “T” represents for trailing side.

In general, the farther away from the wall, the lower is the level of temperature fluctuation within the measured range. It can be seen from Fig. 9a that the effect of rotation is not obvious at the upstream probably due to the development of the turbulent boundary layer is not high. In the downstream of the channel in Fig. 9b, there is a significant difference in the temperature fluctuation distribution between the leading side and the trailing side. The temperature fluctuation intensity of the trailing side changes little, while the temperature fluctuation intensity of the leading side overall decreases as the rotation number increases. From reference materials^23,24^, Coriolis force has the effect of suppressing flow instability near the leading side and enhancing flow instability near the trailing side. Koyama *et al*.^24^ called the phenomenon that the Coriolis force near the leading side suppresses flow instability as relaminarization. Since flow and heat transfer affect each other, it can be inferred that Coriolis force can also suppress the temperature fluctuation of the leading side. When the rotation number reaches 0.28, the temperature fluctuation level can be reduced by more than half. Comparing the more detailed turbulent characteristics study^25^, with the increase of rotation number, the peak of flow instability near the leading side decreases and the ${y}^{+}$ of the peak position increases. This trend does not occur with temperature fluctuations, which is a difference from velocity fluctuations.

#### Higher-order statistics

**Figure 10:**
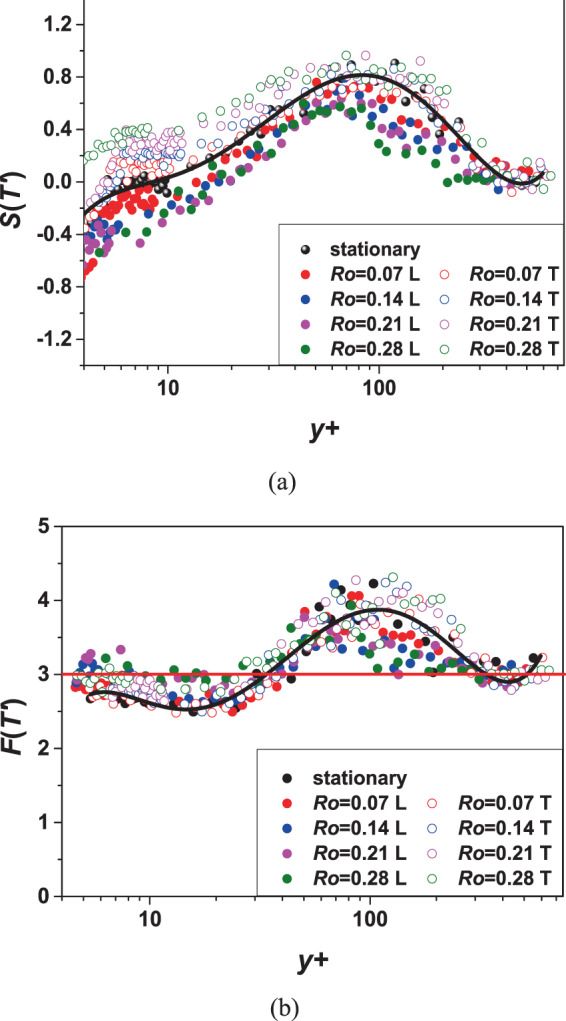
Profiles of the skewness and flatness at location 1.

The high-order statistics of turbulence can reflect more information. The skewness $S(T{\prime} )=\frac{\overline{{(T{\prime} )}^{3}}}{{\overline{{(T{\prime} )}^{2}}}^{\frac{3}{2}}}$ is the third order of the temperature fluctuations, which represents the asymmetry of the probability distribution of the turbulent random fluctuations around the mean values. If the value is positive, it means that the prominence of the probability density function is tilted to the right and the function value is mostly distributed on the right side. If its value is a negative value, it indicates the opposite. Taking location 1 as an example in Fig. 10a, the trend of the skewness is the same under different rotation numbers. The bold solid lines in the figures show the general trend. The rotation effect of the leading side will reduce the skewness while the rotation effect of the trailing side will increase the skewness. This indicates that the rotation effect near the trailing side increases the probability that the temperature fluctuation $T{\prime}$ will take a positive value. The rotation effect near the leading side plays an opposite role. The maximum deviation is about 0.2 compared to the static condition.

The temperature flatness $F(T{\prime} )=\frac{\overline{{(T{\prime} )}^{4}}}{{\overline{{(T{\prime} )}^{2}}}^{2}}$ is the fourth order of the temperature fluctuations, which is depicted in Fig. 10b. From a statistical point of view, this parameter represents the intermittent nature of the probability distribution of turbulent random fluctuations. It is generally believed that if the flatness is greater than 3 (i.e., the flatness of the Gaussian distribution), then the random variable is intermittent. The flatness of the Gaussian distribution is marked with a red horizontal line in the figure. It can be seen that the flatness of the near-wall area and the core area is maintained at around 3. In the area of about $10 < {y}^{+} < 30$, the flatness is less than 3, indicating that the intermittency of this part is weak. While in the area of about $30 < {y}^{+} < 300$, the intermittency is strong due to that the flatness is greater than 3 in this area. The position where the rotation effect has the greatest influence on the flatness is about ${y}^{+}=150$. For temperature fluctuations, the rotation effect at this position causes the intermittent reduction of the leading side and the intermittent enhancement of the trailing side.

### Distributions of streamwise turbulent heat flux profiles

**Figure 11:**
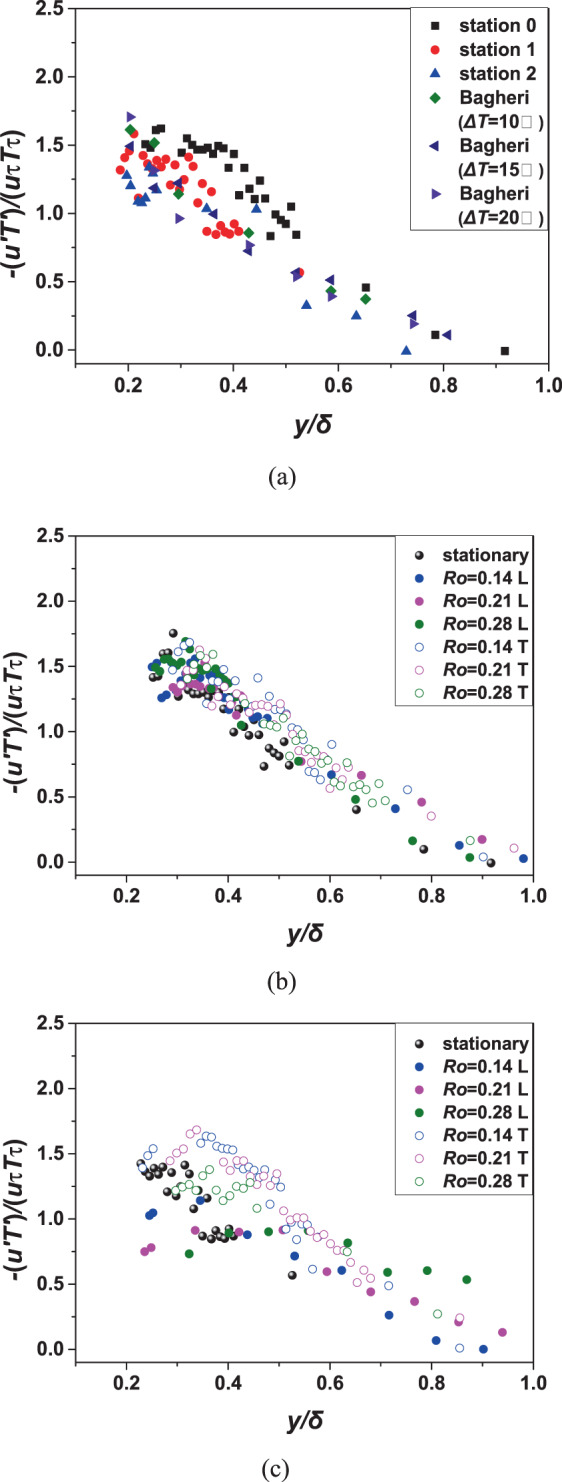
Contrast with the research of Bagheri^16^ (**a**) and streamwise turbulent heat flux profiles under different rotation numbers at location 0 and location 1 (**b,c**).

In addition to the gradient of mean temperature that generates heat flux, the turbulent fluctuations can also generate heat flux, which is called turbulent heat flux. For the case of heated wall, there are usually two types of turbulent heat flux measured: streamwise turbulent heat flux $\overline{u{\prime} T{\prime} }$ and normal turbulent heat flux $\overline{v{\prime} T{\prime} }$. Due to the limitation of test technology, this study only contained the former measurement data. The heat flux $\overline{u{\prime} T{\prime} }$ has been normalized with respect to the friction velocity and temperature and is plotted as a function of $y/\delta$. Here, $\delta$ is the same definition as ${\delta }_{2}$ which is defined in formula (9). The results of the three different temperature cases^16^ are compared with present static results of three different locations in Fig. 11a. It can be seen that these results are roughly consistent. When $y/\delta  < 1$, $\overline{u{\prime} T{\prime} }$ is usually negative, which can be explained simply but not very strict as follows. Under the same working condition, the temperature of the downstream at certain *y* is higher than that of the upstream. When a small disturbance occurs (assuming that $u{\prime}  > 0$), the fluid microbody have a tendency to move downstream compared with time-average velocity, which is equivalent to a negative disturbance in the local temperature and produces a negative $T{\prime}$. For the same reason, a negative $u{\prime}$ produces a positive $T{\prime}$.

The rotation effect on the streamwise heat flux can be derived from Fig. 11b and c (*x*/*D* = 4.06 and 5.31). It can be seen that at a position where $y/\delta$ is approximately equal to 0.4, the turbulent heat flux is equal to the product of the friction velocity and the friction temperature. The dimensionless heat flux shows a linear decrease trend in the boundary layer. When $y/\delta$ achieves 1, Dimensionless heat flux approximately reduces to 0. It can be seen from Fig. 11b that at the upstream, the dimensionless heat flux under different *Ro* is similar to that under static conditions. As for Fig. 11c, the downstream turbulent heat flux especially that of the leading side is reduced overall and is greatly affected by rotation numbers. When *Ro* = 0.28 at the leading side, the dimensionless heat flux (which is expressed with solid green circles) decreases very slowly. There is a significant difference from the laws of other curves.

## Conclusion

This experiment measured the instantaneous tempera-ture and velocity field synchronously in non-isothermal turbulent boundary layer in a rotating channel with a parallel-array hot-wire probe. The Reynolds number based on the bulk mean velocity and hydraulic diameter is 19000, and the rotation numbers are 0, 0.07, 0.14, 0.21 and 0.28. Three streamwise locations(*x/D* = 4.06, 5.31, 6.56) were investigated. According to the results, the conclusions can be drawn as follows:

1. By comparing with other researcher’s velocity and temperature obtained in the turbulent boundary layer, it can be confirmed that the home-made measurement system can be achieved to measure velocity and temperature in a non-isothermal turbulent boundary layer at rotating conditions, and the data obtained in current work is credible.
2. For the leading side, rotation has little effect on dimensionless temperature ${T}^{+}$ at the upstream. At the downstream in, the ${T}^{+}$ with different *Ro* shows a significant difference after ${y}^{+}$ is greater than 30. As for the trailing side, the rotation effect is more obvious and makes the dimensionless temperature profiles lower than that under static conditions. When the rotation number is small, the rotation effect near the trailing side can be ignored.
3. At the downstream, the temperature fluctuation intensity near the trailing side changes little with the change of rotation numbers. While near the leading side, the temperature fluctuation intensity decreases as the rotation number increases. It can be inferred that Coriolis force can suppress the temperature fluctuation of the leading side which is called relaminarization. The rotation effect reduces the skewness and flatness of the leading side and increases those of the trailing side.
4. The dimensionless heat flux shows a linear decrease trend in the boundary layer. Relative to the upstream, the downstream turbulent heat flux especially that of the leading side is reduced overall and is greatly affected by rotation numbers.
